# Plasticity for axolotl lens regeneration is associated with age‐related changes in gene expression

**DOI:** 10.1002/reg2.25

**Published:** 2014-10-12

**Authors:** Konstantinos Sousounis, Antony T. Athippozhy, S. Randal Voss, Panagiotis A. Tsonis

**Affiliations:** ^1^ Department of Biology and Center for Tissue Regeneration and EngineeringUniversity of DaytonDaytonOHUSA; ^2^ Department of Biology and Spinal Cord and Brain Injury Research CenterUniversity of KentuckyLexingtonKY40503USA

**Keywords:** Axolotl, lens, microarray, newt, regeneration

## Abstract

Mexican axolotls lose potential for lens regeneration 2 weeks after hatching. We used microarrays to identify differently expressed genes before and after this critical time, using RNA isolated from iris. Over 3700 genes were identified as differentially expressed in response to lentectomy between young (7 days post‐hatching) and old (3 months post‐hatching) axolotl larvae. Strikingly, many of the genes were only expressed in the early or late iris. Genes that were highly expressed in young iris significantly enriched electron transport chain, transcription, metabolism, and cell cycle gene ontologies, all of which are associated with lens regeneration. In contrast, genes associated with cellular differentiation and tissue maturation were uniquely expressed in old iris. Many of these expression differences strongly suggest that young and old iris samples were collected before and after the spleen became developmentally competent to produce and secrete cells with humoral and innate immunity functions. Our study establishes the axolotl as a powerful model to investigate age‐related cellular differentiation and immune system ontogeny within the context of tissue regeneration.

## Introduction

Regenerative ability varies across organs, developmental stages, and species. However, one generality that has been noted for highly and lowly regenerative vertebrates is that regenerative ability tends to decrease with age (Sousounis et al. 2014). Fetal and larval forms tend to possess an ability to regenerate tissue in a scar‐free manner while adults, and especially mammals, show minimal potential for regeneration. These patterns suggest that regenerative ability is associated with age‐related changes in cells that form tissues and organs, as well as maturation of systems that broadly regulate development and physiology (Seifert & Voss 2013). Exceptions include lens regeneration in adult newts (Eguchi et al. 2011) and fin regeneration in zebrafish (Itou et al. 2012).

Some amphibians are capable of regenerating their lens through a process called transdifferentiation. During embryonic development of salamanders, the lens is formed by invagination of the surface ectoderm, which later differentiates into cornea (Wolff 1895; Freeman 1963; Suetsugu‐Maki et al. 2012). In contrast, progenitor cells that regenerate lens after lentectomy derive from the iris, which has a neural origin (Fuhrmann 2010; Graw 2010). Thus, transdifferentiation refers to a special type of regeneration where progenitor cells from a different tissue are the source of the regenerate.

The adult red‐spotted newt (*Notophthalmus viridescens)* has long served as the primary salamander model for studies of transdifferentiation and lens regeneration. Soon after lentectomy, pigment epithelial cells (PECs) of the dorsal and ventral iris dedifferentiate; however, only PECs from the dorsal iris contribute progenitors for lens regeneration (Sato 1940). For many years, the axolotl (*Ambystoma mexicanum*) was thought to lack the newt's lens regenerative potential; however, it was recently shown that axolotls can in fact regenerate lens from dorsal and ventral iris PECs during early larval development (Suetsugu‐Maki et al. 2012). But, after approximately 28 days of post‐hatching development, axolotl larvae lose the ability to regenerate lens. Thus, the axolotl provides an important new model to identify age‐related changes in gene expression that correlate with regenerative ability. In this study, we used microarray analysis to identify gene expression differences between irises collected from 7‐day post‐hatching larvae (referred to as young) and 3‐month‐old larvae (referred to as old). We collected tissues post‐lentectomy to sample regeneration‐associated transcripts from young iris and transcripts associated with a non‐regenerative response in old iris. The genes that were expressed differently between young and old axolotl larvae reveal age‐related differences in transcription, metabolism, cell proliferation, differentiation, and immune response. We report further insights by comparing genes identified between young and old axolotl iris to genes that were identified recently from dorsal and ventral irises of newts (Sousounis et al. 2013).

## Results

### Gene expression during axolotl lens regeneration

**Figure 1:**
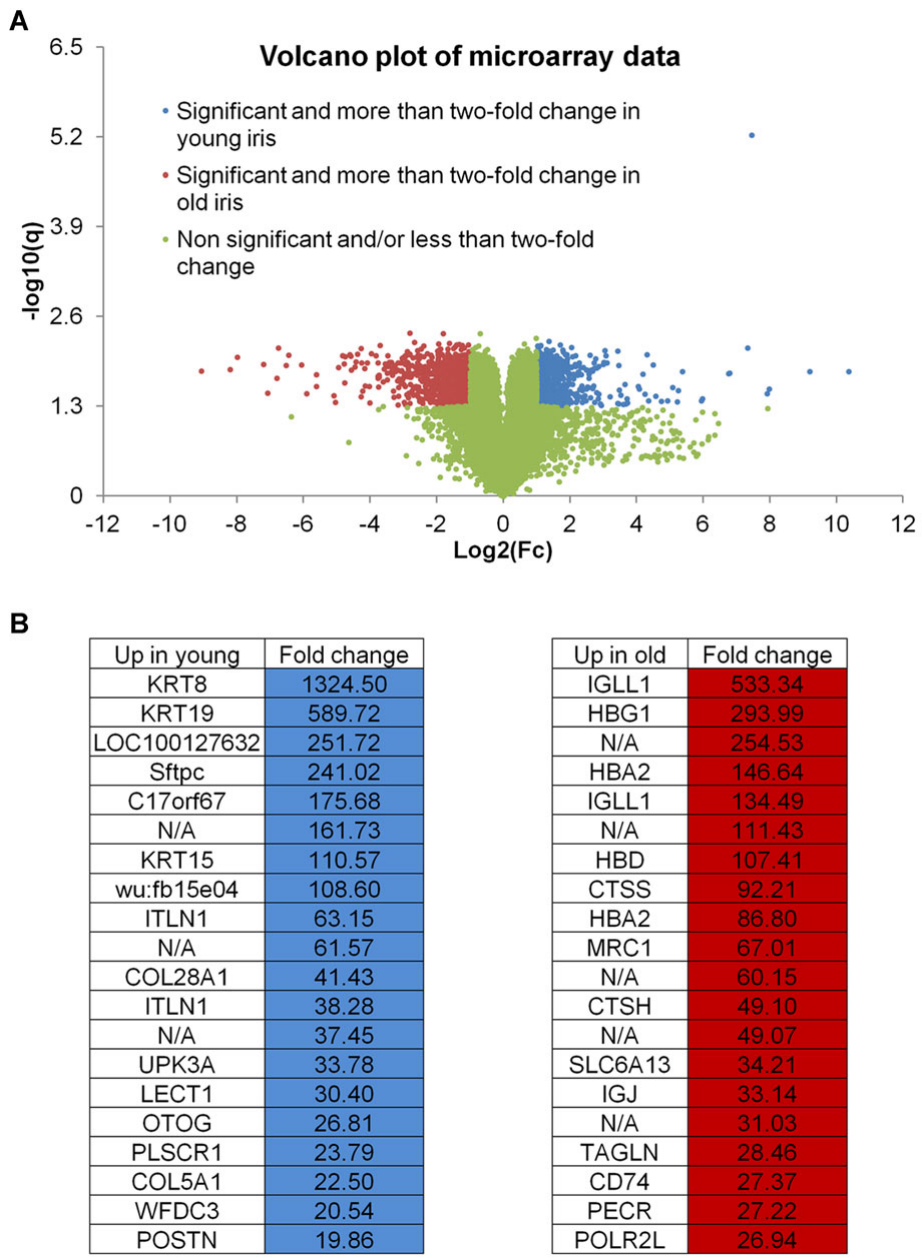
Microarray gene expression during axolotl lens regeneration. (A) Volcano plot of all the probe sets of the microarrays. Probe sets are color‐coded based on the significance and the fold change between the samples. (B) Highly upregulated genes in young and old iris samples. N/A, not applicable.

Young and old axolotl larvae were lentectomized and 6 hours later whole iris rings were isolated for RNA extraction and Affymetrix microarray analysis. A total of 3751 probe sets (i.e. genes) were identified as statistically, differentially expressed between the young and old iris samples, and, of these, 1572 registered a > 2‐fold difference in expression (Table S1). Approximately half of the differentially expressed genes were more expressed in young iris samples (*N* = 1809) and thus the remainder were more expressed in old iris (*N* = 1942) (Fig. 1A). Strikingly, many of the upregulated genes were highly differentially expressed between samples. For example, *krt8*, *krt19*, *sftpc*, *itln1*, and *col28a1* were 1324 to 41 times more abundant in young iris than old. Moreover, *igll1*, *hbg1*, *hba2*, *ctss*, *mrc1*, and *slc6a13* were 533 to 34 times more abundant in old iris (Fig. 1B). Examination of expression estimates for all of the genes listed above, and 168 additional genes, suggests that they were only expressed in one of the iris samples. Affymetrix probe sets for these genes registered low, mean expression values for one of the samples, values that did not eclipse an empirically determined threshold for defining absence of expression (see Materials and Methods). Thus, these results show fundamental differences in transcription between young and old iris, with > 100 genes expressed in one sample but not the other. In addition to the genes listed above, we note that additional keratins (*krt15*, *krt18*) and collagens (*col5a1*, *col12a1*, and *col29a1*), and a biomarker of cell proliferation (*shcbp1*), were only expressed in regeneration competent young iris.

### Gene Ontology (GO) enrichment analysis

**Figure 2:**
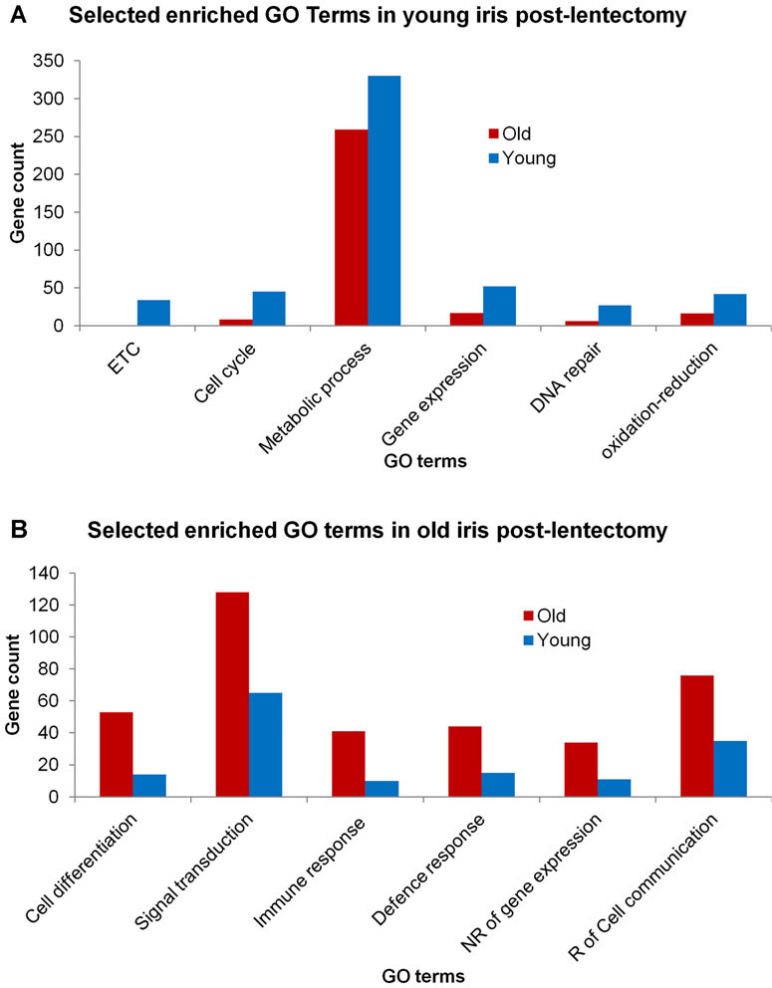
Selected enriched GO terms in axolotl samples. (A) Selected enriched GO terms in young iris samples (*q* < 0.05). (B) Selected enriched GO terms in old iris samples (*q* < 0.05). Bars indicate the number of genes found with the corresponding GO term.

Statistically significant genes (*q* < 0.05) that minimally exhibited a 2‐fold difference between the iris samples were selected for Gene Ontology (GO) enrichment analysis. The genes identified from young iris samples enriched GO terms associated with regulation of gene expression, electron transport chain, cell cycle, DNA repair, oxidation−reduction process, and metabolic process (*q* < 0.05, Fig. 2A, Tables 1, 2 and S2). The genes that significantly enriched these terms are predicted to regulate transcription (*ccnh*, *cdk7*, *gtf2a2*, *taf5*, *taf9*, *taf13*, and *taf15*), splicing (*lsm1*, *lsm3*, *lsm5*, *lsm6*, *lsm7*, *phf5a*, *snrpa1*, *snrpd2*, and *snrpd3)*, ATP production (*ndufa1−7*, *ndufa12*, *ndufb2*, *ndufb4−8*, *ndufc2*, *ndufs5*, *ndufs6*, *ndufv2*, and *ndufv3)*, intracellular protein levels (*psma3*, *psma4*, *psma7*, *psmb2*, *psmb7*, *psmd12*, and *psmd8)*, DNA replication (*chaf1a*, *gins1*, *pole2*, *dbf4*, *rpa2*, and *tyms*), DNA repair (*nsmce1*, *rad51*, *rad51ap1*, *trip13*, and *rpa2)*, and chromosome segregation (*cdc27*, *cdca8*, and *kif20a*). Overall, these expression results suggest that young iris was metabolically more active and proliferative than old iris, as would be expected if the former were initiating a larval dedifferentiation response (Reyer 1982). We note that these expression differences were quantitative and not absolute as the genes listed above were also expressed in old iris, at significantly lower levels, however.

**Table 1:** Genes related to gene expression that were found to be significantly upregulated in the young axolotl iris

Function							
Transcription	CCNC	CDK7	E2F4	MTERF	POLR2K	TAF13	TAF9
	CCNH	CDK8	GTF2A2	POLR1D	POLR3F	TAF5	TAF15
RNA processing	CPSF3	HNRNPM	LSM5	PHF5A	SNRPD2	SRSF5	NCBP1
	EXOSC6	LSM1	LSM6	RNGTT	SNRPD3	WDR77	SSU72
	FUS	LSM3	LSM7	SNRPA1			
Translation	EIF4E	MARS	RPL29	RPL34	RPL6	RPS24	SARS2
	IARS2	NARS	RPL31	RPL38	RPS21		
Protein processing	BAX	PSMA3	PSMA7	PSMB7	PSMD14	RBX1	SEC61G
	FBXO6	PSMA4	PSMB2	PSMD12	PSMD8	SEC13	SPCS3
	PDIA6						

**Table 2:** Genes related to electron transport chain, cell cycle, and DNA repair that were found to be significantly upregulated in young axolotl iris

Function							
Electron transport chain	ATP5D	ATP5L	ETFA	NDUFA4	NDUFB4	NDUFC2	UQCR10
	ATP5E	COX6A1	NDUFA1	NDUFA5	NDUFB5	NDUFS5	UQCR11
	ATP5I	COX6C	NDUFA12	NDUFA6	NDUFB6	NDUFS6	UQCRB
	ATP5J	COX7B	NDUFA2	NDUFA7	NDUFB7	NDUFV2	UQCRQ
	ATP5J2	COX7C	NDUFA3	NDUFB2	NDUFB8	NDUFV3	
Cell cycle	CCNH	DYNLL1	KRT18	NUF2	PSMA3	PSMD14	SEC13
	CDC26	E2F4	LIN9	NUP85	PSMA4	PSMD8	SKA2
	CDC27	E2F8	MCTS1	ORC6	PSMA7	PTTG1	SSNA1
	CDCA8	GINS1	MRPL41	PDCD2L	PSMB2	RAB2A	TOP2A
	CDK7	GORASP2	NDC80	PFDN1	PSMB7	RFC2	TXLNG
	CHAF1A	KIF20A	NOLC1	POLE2	PSMD12	RPA2	TYMS
	DBF4	KIF23					
DNA repair	ACTL6A	EYA2	INO80C	POLG2	PTTG1	RBX1	TOP2A
	CCNH	FBXO6	NEIL3	POLR2K	RAD17	RFC2	TRIP13
	CDK7	GTF2H5	NSMCE1	PRMT6	RAD51	RPA2	TYMS
	CHAF1A	HMGA2	POLE2	PSMD14	RAD51AP1		

A different set of GO terms were identified for old iris samples – immune response, defense response, cell communication, signal transduction, negative regulation of gene expression, and cell differentiation (*q* < 0.05, Fig. 2B, Tables 3, 4 and S3). Many of the genes that enriched these terms were only expressed in old iris, including factors associated with innate immunity (*cd74*, *ctsh*, *ctss*, *cfd*, *ctsg*, *igj*, *ighm*, *igll1*, *igsf1*, *f13b*, *pros1*, *ccl19*, *tgfb2*, *mrc1*, *enpp2*, and *ighm*), and cellular growth and differentiation (*hes5*, *fgf13*, *edar*, *vwc2*, *adfp*, *cntnap2)*. Many additional genes associated with cellular differentiation were expressed more highly in old iris than young, including *cdh2*, *dner*, *gpm6a*, *ndrg2*, *ndrg4*, *numb*, *pirin*, *wisp1*, *notch*, *bmp2*, *bmp7*, *rb1*, *atf1*, *atf5*, *aft6*, *jag1*, *fgfbp3*, *fgfr1*, *kit*, *ctnnd1*, *smad7*, *igfbp3*, *igfbp6*, *hgf*, *tgfbi*, *tgfb1*, *ctgf*, *procn*, *igfals*, and *lhx2*. Overall, the identified genes clearly indicate that a post‐lentectomy immunological response was induced in old iris, a response that was not observed in young iris. In addition, in comparison to young iris, the results suggest that old iris was relatively more differentiated and presented less potential for cell proliferation. Indeed, negative regulators of DNA synthesis (*enosf1*) and cell cycle progression (*mll5*, *kiss1r)* were expressed more highly in old iris.

**Table 3:** Genes related to cell differentiation that were found to be significantly upregulated in the old axolotl iris

A2M	CBLN1	CTGF	EXT2	IRF1	KRT8	NUMB	SLC7A11	TRAPPC9
ARHGAP24	CCL19	CTSV	FHL1	IRF8	MGMT	PIR	STEAP4	UHRF2
B2M	CDH2	DNER	GNA12	JUN	MSI1	PPDPF	TDRKH	ZFP36L1
BMP2	CHRDL1	EDAR	GPM6A	KIT	NDRG2	PSAP	TGFB1	ZFPM2
BNIP3	CREBL2	EPAS1	HERC4	KMT2E	NDRG4	SEMA4A	TGFB2	ZSCAN2
CAMK4	CREM	ERAP1	HES5	KRT19	NOTCH1	SKIL	TMEM176B	

**Table 4:** Genes related to immunity which were found to be significantly upregulated in old axolotl iris

ADCY2	CCL19	CTSG	ENPP2	IGJ	KIT	PCBP2	PROS1	TGFB2
ADCY3	CD59	CTSH	ERAP1	IGLL1	MR1	PLD2	SFTPD	TLR2
APOA4	CD74	CTSS	FTH1	IRF1	NFIL3	POLR2L	SPPL2B	TRIM11
B2M	CHIT1	CXCL10	HLA‐E	IRF8	NOTCH1	PRF1	TGFB1	TRIM35
CAMK4	CLU	ECM1	HSP90AA1	JUN				

### Validation with qPCR

**Figure 3:**
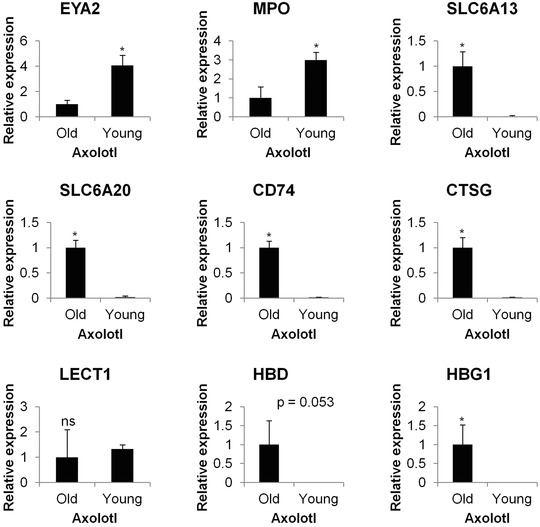
Gene expression validation with qPCR. EYA2, MPO, SLC6A13, SLC6A20, CD74, CTSG, LECT1, HBD and HBG1 gene expression was found with qPCR. Bars indicate the average of three independently collected iris samples of the corresponding axolotls. Error bars indicate standard deviation. Asterisk (*) indicates statistically significant with 95% confidence intervals (*P* < 0.05) determined by *t*‐test for independent samples. Equal variances were determined with Levene's test. ns, not significant.

Several genes were selected for independent validation of microarray expression estimates using quantitative polymerase chain reaction (qPCR). Using biological replicates, qPCR yielded highly similar estimates to those obtained by microarray (Fig. 3). Two of three genes (*eya2* and *mpo*, but not *lect1*) that were estimated as highly differentially expressed in young iris were validated, as were all six genes that were deemed as only expressed in old iris (*slc6a13*, *slc6a20*, *cd74*, *ctsg*, *hbd*, and *hbg1)*. The qPCR estimates for *lect1* did not reveal a significant difference between young and old iris, as was suggested by the microarray analysis. Overall, qPCR validated all but one of the microarray estimates (Fig. 3).

### Comparison of gene expression patterns between axolotl and newt lens regeneration

**Figure 4:**
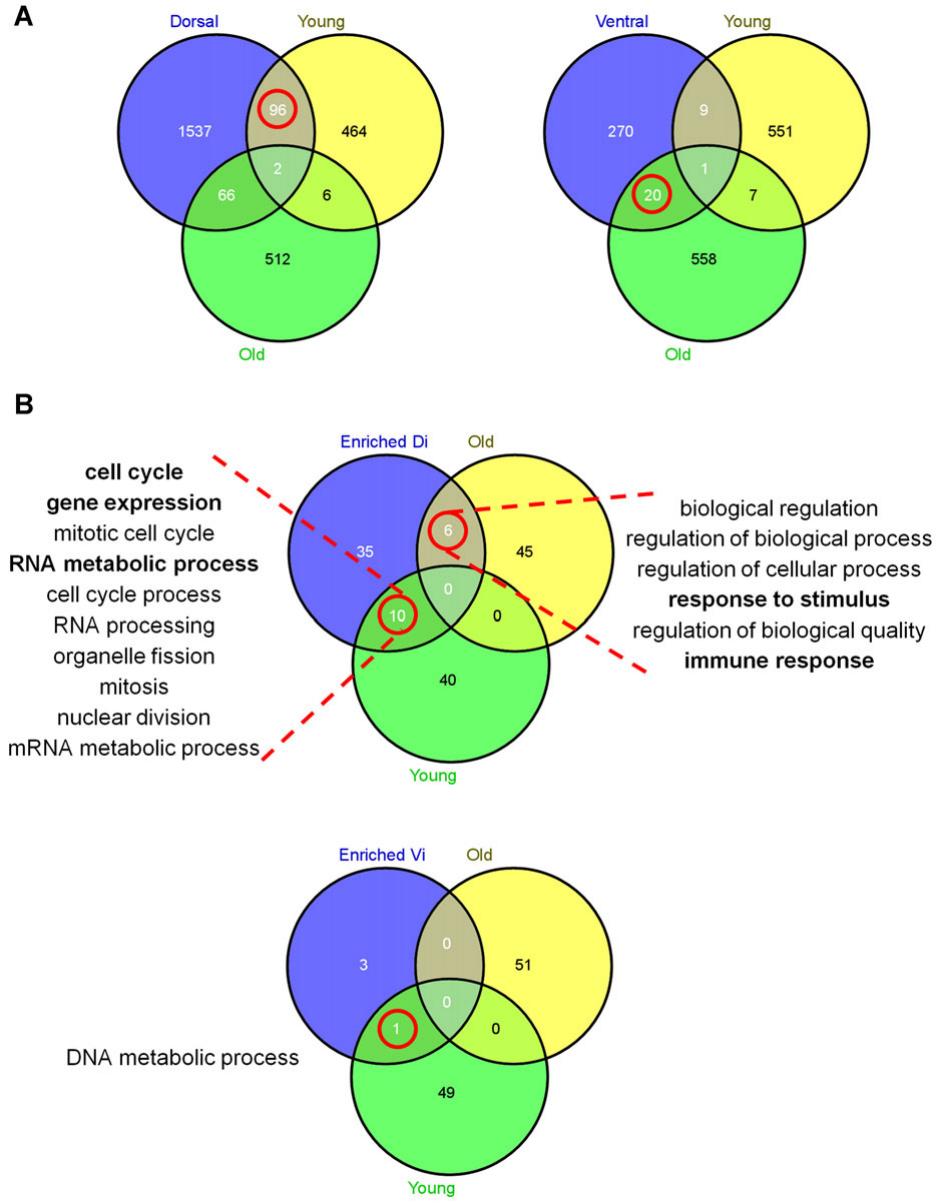
Comparative transcriptomics between axolotl and newt lens regeneration. (A) Newt genes found to be upregulated at least 2‐fold in dorsal iris compared with ventral iris during lens regeneration, and vice versa, are compared with genes found to be upregulated at least 2‐fold in young and old axolotl iris during lens regeneration. (B) GO terms found to be enriched in newt dorsal or ventral iris samples during lens regeneration are compared with GO terms found to be enriched in axolotl young or old iris samples during lens regeneration. The common GO terms are presented adjacent to the Venn graph as indicated with dotted red lines. Highly discussed GO terms are indicated in bold. All comparisons are presented as Venn graphs. Red circle indicates the highest similarity for each comparison.

Recently, RNA sequencing was used to identify genes expressed differently between regeneration competent dorsal and regeneration incompetent ventral iris during newt lens regeneration (Sousounis et al. 2013). We compared genes from Sousounis et al. (2013) that exhibited a > 2‐fold difference between dorsal and ventral iris at 4 or 8 days post‐lentectomy (DPL) to genes identified as significant in our study. We found greater overlap of significant genes and enriched GO terms between regeneration competent newt dorsal iris and young axolotl iris than regeneration incompetent newt ventral iris and old axolotl iris (Fig. 4). In particular, GO terms for transcription, cell cycle, and metabolic process were identified in common between newt dorsal iris and young axolotl iris, while innate immune responses were identified in common between newt dorsal iris and old axolotl iris. The 96 genes that were expressed more highly in young axolotl and newt dorsal iris than old axolotl and newt ventral iris provide important new candidates for functional studies (Table 5). Also, 20 genes that were commonly upregulated in regeneration incompetent irises in both species implicate these as candidate inhibitors of regeneration. We discuss several genes identified from this bioinformatics analysis below.

**Table 5:** Genes found to be upregulated in both newt and axolotl lens regeneration in regeneration competent or incompetent iris

Function	Gene upregulated in regeneration competent iris						
Transcription	CIRH1A	ENY2	POLR1D	RBBP7	ZNF182	ZNF451	
RNA processing	CPSF3	MPHOSPH10	PDCD11	PNPT1	SNRPA1		
Translation	C12orf65	MRPL19	MRPL41	MRPL53	MRPS27	MRPS28	QRSL1
Protein processing	CRELD2	PFDN4	TIMM13	TIMM8A	TIMM9	VPS13A	WDR77
	P4HA1	PHPT1					
Electron transport chain	ATP5D	UQCRQ	CMC1	COX16	ETFA	NDUFS5	
Metabolic process	BCAT1	CYP51A1	FDPS	LSS	PXDN	ROMO1	SQLE
	CYP26A1	DHRS12	GPT2	PTS	RDH13	SLC35B1	TPMT
	CYP2C8	DPH5	GSS				
Extracellular matrix	COL12A1	DPT	HTRA1	MXRA5			
Cell cycle	CDC27	DSCC1	KIF11	LIN9	NUF2	PRC1	RPA2
	CDCA8	GINS1	KIF20A	NCAPG2	PBK	RCC1	RPS6KB1
	CHAF1A	HAUS1	KIF23	NDC80	PDCD2L	RNASEH2A	TOP2A
	DBF4						
Other	BAX	C19orf60	C7orf25	LRRC32	PRKRIR	RP9	TMPO
	C11orf10	C6orf162	CASP3	NSMCE1	RHOT1	SSNA1	TOR1AIP1
	**Gene upregulated in regeneration incompetent iris**						
	ACTA2	CPAMD8	KDM4C	NR2F1	SLC1A3	STXBP4	ZNF510
	CHRDL1	HSPA8	LAMA2	NRCAM	SLC6A13	TRIM11	ZNFX1
	CHST11	KCNMB2	LAMB2	RGMB	SLIT2	TTC17	

**Figure 5:**
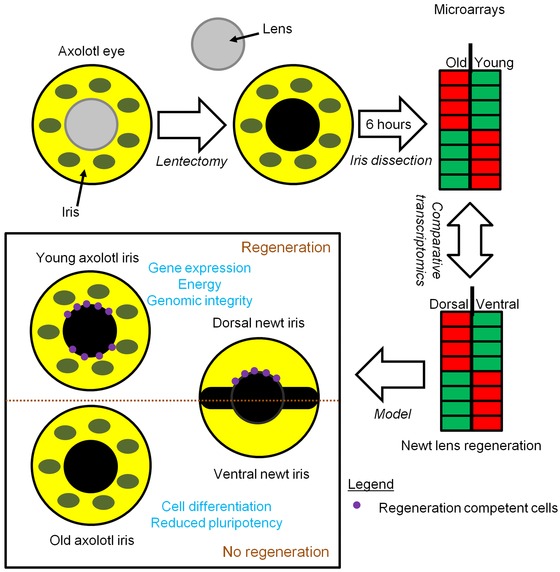
Proposed model for lens regeneration in newts and axolotls. Lens regeneration competent tissues (young axolotl iris and newt dorsal iris) have potent cells that can be activated and carry out similar events leading to transdifferentiation. Newt ventral iris and old axolotl iris contain more differentiated cells that lack pluripotency and the ability to be activated post‐lentectomy.

## Discussion

Regenerative ability varies greatly among vertebrates but is generally much higher during early life stages (Seifert & Voss 2013; Sousounis et al. 2014). In this study the early transcriptional response of iris to lentectomy was compared between young and old axolotl larvae that differed in regeneration competence. Using only three replicate Affymetrix GeneChips per treatment, > 3700 differentially expressed genes were identified statistically and many of these genes exhibited 10–100‐fold expression differences between treatments. The many highly differentially expressed genes identified in our study are probably explained by the presence and absence of different cell types between young and old iris tissue and age‐related changes in cellular differentiation. We discuss both of these explanations below, and then discuss new gene expression insights that were gained by comparing our results with those obtained recently from newts.

### Ontogeny of immunity correlates with loss of regenerative ability in the Mexican axolotl

In axolotls, the spleen is the organ where erythrocytes, lymphocytes, and thrombocytes are produced and released into the blood (Charlemagne 1972). Although the axolotl spleen begins to differentiate during the later stages of embryonic development, maturation is not completed until larvae reach 2–3 months of age. Immunoglobulin M (IgM*)* synthesizing lymphocytes are first observed approximately 35 days post‐hatching in the spleen, and then 56–70 days after hatching in serum (Fellah et al. 1989). This explains why genes encoding heavy and light chain components of IgM (*ighm*, *igj*, *igll1*, *igsf1*) and hemoglobin gamma A (*hbg1*) were highly expressed in the old iris samples but not in young iris samples. The axolotls that provided the older iris tissue were approximately 84 days post‐hatching and thus had circulating lymphocytes and erythrocytes. In support of this explanation, genes associated with immune cells and system responses were uniquely expressed in old iris, including genes associated with macrophages (*mrc1*, *ctsh*, *ctsg*), basophils/mast cells (*hdc*), and B‐cells (multiple immunoglobulins, *lrrc8d*), and processes ranging from coagulation (*f13b*, *pros1)*, lymphocyte homing and migration (*ccl19*, *enpp2*, *wasf3*), complement (*cfd*), and antigen presentation and processing (*cd74*, *ctss*). These gene expression results are consistent with the above timeline for axolotl spleen development (Fellah et al. 1989) and hemoglobin switching (Page et al. 2010), and clearly show that some humoral and immunological gene expression responses to injury change with aging. These differences between the early and old iris are absolute and robust; if no B‐cells are present in a tissue sample, no B‐cell‐associated transcripts will be measured. In future studies, it will be important to more broadly sample the larval period, as such a design could better resolve age‐related changes in gene expression that are quantitative in nature. Such a design would be informative for understanding how injury and non‐injury responses change with aging, perhaps comparing in parallel regeneration competent and incompetent tissues. Our results suggest that such a study could be readily performed using axolotls, and such a study would probably provide important new insights about the maturation of tissues and physiological systems within the context of tissue regeneration.

### Cellular differentiation also correlates with loss of regenerative ability

Cells differentiate and tissues mature as an organism ages. In general, cells become more differentiated and less stem‐like with aging and may show lower potential for dedifferentiation, cell cycle re‐entry, and patterning (Sousounis et al. 2014). During the aging process of vertebrates with low potential for regeneration (e.g., mammals), cells may differentiate toward fates that are more appropriate for tissue repair and less permissive for regeneration. Independent of immune system function, our results support the idea that axolotl iris differentiates with aging to a point that it is no longer capable of dedifferentiation. Indeed, we identified a number of regulators/biomarkers of cellular differentiation that were more highly expressed in old iris, including *notch*, *bmp2*, *bmp7*, and *rb1*. Interestingly, Grogg et al. (2005) were able to induce lens regeneration by inhibiting bone morphogenetic protein 4 (BMP4) and BMP7 expression in regeneration incompetent ventral iris PECs of the newt, but similar treatments did not induce lens regeneration in axolotl (Grogg et al. 2005). This suggests that age‐related changes in regenerative ability may involve transcriptional changes across multiple signaling pathways; and such changes may specify non‐regenerative cellular phenotypes. Two lines of evidence support this idea. (1) We observed genes expressed in young iris that promote cell proliferation and genes expressed in old iris that function to restrict DNA synthesis and cell proliferation. For example, *enosf1* and *ctnp2* were highly expressed in old iris. *enosf1* encodes an anti‐sense transcript that downregulates thymidylate synthase, an enzyme that functions in thymine biosynthesis, while *ctnp2* catalyzes the rate‐limiting step in cytosine synthesis. These patterns suggest that lentectomy causes an imbalance of nucleotide precursors in old iris, a molecular pathology that is not optimal for supporting cell proliferation. (2) Johnson (2013) recently showed that the expression of genes for cell proliferation and collagen synthesis declined with aging in axolotl brain; both of these patterns were observed in our study. Genes that are permissive for lens regeneration are expressed highly early in the larval period but are gradually or suddenly downregulated during development. We note that the loss of lens regenerative plasticity in the Mexican axolotl occurs after the first 28 days of post‐hatching development, which associates with not only the initiation of immune system function but also gonadal differentiation (Gilbert 1936). Disentangling the effects of local and peripheral factors on regenerative capacity can be tested by grafting young iris cells into regeneration incompetent older eyes, or by moderating the immune response of older axolotls, as was done recently in a study of macrophage function during axolotl limb regeneration (Godwin et al. 2013).

### Identification of new candidate genes for lens regeneration

Finally, we compared lists of genes that were compiled from two different lens regeneration models. We compared genes that were identified as differentially expressed between young and old axolotl iris to genes identified as differentially expressed between dorsal and ventral regions of the adult newt iris. The objective was to determine if gene expression was similar for regeneration competent and incompetent samples, even though they were derived from different species and experimental paradigms (the effect of aging versus patterning on regenerative ability). Somewhat surprisingly, given low power to detect homologous expression results between lowly replicated studies that derive expression estimates from different technologies, and given reports indicating axolotls and newts employ different mechanisms to accomplish the same regenerative outcome (e.g., Sandoval‐Guzman et al. 2014), we identified common gene expression responses. In particular, genes associated with cholesterol metabolism (*cryp51a*, *lss*, *fdps*, *sqle*), retinoic acid synthesis (*rdh13*, cyp26a), and mitosis/regulation of cell proliferation (e.g., *dscc1*, *pbk*, *lin9*, *romo1)* were identified for regeneration competent axolotl and newt iris. Thus, genes identified from regeneration competent iris, and presumably dedifferentiating and proliferating PECs, probably comprise a conserved regulatory network underlying transdifferentiation (Fig. 5). Upregulation of *cyp26a* in regeneration competent iris is interesting because it acts to attenuate retinoic acid signaling, a metabolite required for lens regeneration in adult newt and *Xenopus* (Thomas & Henry 2014). Among genes that were expressed in common between regeneration incompetent axolotl and newt iris, we note repressive axon guidance molecules (*slit2*, *rgbm*), neurotransporters (*slc1a3*, *slc6a13)* indicating possible roles in cell to cell attraction or repulsion (Kim et al. 2014), and *nr2f1*, a transcription factor that specifies neural cell fates and negatively regulates retinoic acid signaling (Neuman et al. 1995; Yamamizu et al. 2013). These genes further support the idea that regeneration incompetent iris is associated with higher expression of differentiation markers. Overall, our comparative analysis shows that regenerative ability of salamander iris is associated with cholesterol biosynthesis and retinoic acid synthesis and signaling.

## Materials and Methods

### Animals and operations

*Ambystoma mexicanum* embryos and larvae were purchased from the Ambystoma Genetic Stock Center in Lexington, KY. The young iris samples were collected from individuals that were raised from embryos to 7 days post‐hatching. For older animals, 3‐month‐old axolotl larvae (3–5 cm) were purchased. Axolotls were anesthetized in 0.1% (w/v) ethyl‐3‐aminobenzoate methanesulfonic acid (MS222; Sigma‐Aldrich, St. Louis, MO, USA) in phosphate‐buffered saline. Using a sharp scalpel, an incision was introduced in the cornea. Lenses were removed with fine forceps ensuring that lens fibers and capsules were removed without damaging adjacent tissues. Six hours post‐lentectomy, axolotls were anesthetized in MS222 and whole eye balls were removed in calcium‐ and magnesium‐free Hanks’ solution where they were dissected according to the method of Bhavsar et al. (2011). Briefly, a fine scalpel was used to make a hole in the eye and scissors were used to separate the anterior and posterior eye parts. Iris pieces were separated from neural retina and cornea and placed in Eppendorf tubes with RNAlater solution (Life Technologies, Grand Island, NY, USA). Three microarray replicates were created for the young and old iris samples by pooling tissues from 11 7‐day‐old and four 3‐month‐old axolotl larvae, respectively. This same procedure was used to create a second, independent group of replicates for qPCR.

### RNA extraction, reverse transcriptase reaction and qPCR

Methods that were used to isolate RNA, synthesize cDNA, and perform qPCR are detailed in Sousounis et al. (2013). qPCR conditions were optimized initially using PCR and gel electrophoresis. Primer sequences and qPCR settings are listed in Table S4. Gene expression estimates were calculated relative to the expression of a housekeeping gene (*eef1a1*).

### Microarrays

The University of Kentucky Microarray Core Facility performed microarray analysis according to standard Affymetrix protocols. All RNA samples were quantified using an Agilent BioAnalyzer. RNA expression profiling was conducted using custom Amby_002 microarrays (Huggins et al. 2012). The six RNA samples were labeled and hybridized to independent microarray GeneChips and scanned. Background correction, normalization, and expression summaries were accomplished using the robust multi‐array average (RMA) algorithm (Irizarry et al. 2003).

### Statistical analysis

To identify significant genes between young and old iris samples (microarray and qPCR analyses), *t*‐tests were performed assuming unequal variances and independent samples. Multiple testing used a false discovery rate cutoff of 0.05 (Benzamini & Hochberg 1995) and was performed by calculating *q*‐values for individual probe sets. This was accomplished by dividing the number of probe sets expected to be false positives at or below the *P* value for a given probe set by the total number of probe sets detected at or below that *P* value. Genes with *q* < 0.05 were considered significant. The method described by Buechel et al. (2011) was used to identify significant probe sets that were expressed in one sample but not the other. Briefly, a probe set was considered non‐expressed if its expression estimate failed to exceed a threshold value that was identified in the saddle region of each array's signal intensity histogram. Gorilla software was used to identify significantly enriched GO terms for significant genes that showed > 2.0‐fold difference in expression between young and old iris samples (Eden et al. 2009). GO terms with *q* < 0.05 were considered significant.

### Comparative transcriptomics

Newt genes that showed > 2‐fold difference in expression between dorsal versus ventral iris (Sousounis et al. 2013) were compared to significant genes identified by contrasting young and old axolotl iris samples. Newt and axolotl genes with the same human gene annotation were assumed to be orthologous and presumptive gene functions were deduced from the literature. Enriched GO terms were also compared between the species. Venn graphs were created using VENNY (http://bioinfogp.cnb.csic.es/tools/venny/index.html).

## Supporting information

Additional Supporting Information may be found in the online version of this article at the publisher's website:

**Table S1**. Microarray gene expression data and statistical analysis.

**Table S2**. Gene Ontology enrichment analysis of young axolotl iris samples.

**Table S3**. Gene Ontology enrichment analysis of old axolotl iris samples.

**Table S4**. List of primers used for qPCR.
